# 

**Published:** 1913-04-26

**Authors:** 


*Just Published.* *New and Enlarged Edition.*

Vicious Circles in Disease.

WITH NUMEROUS ILLUSTRATIONS,

BY

JAMIESON B. HURRY, M.A., M.D.(Cantab.),

*Formerly Medical Officer, University College, Reading.*

"This aspect of Medicine is one which no Practitioner of the *Ars Medendi* can afford to neglect."

Sir Clifford Allbutt, in *Nature*, May 18, 1911.

7/6 net.

J. & A. CHURCHILL, 7 Great Marlborough Street, W.

### Appointments.

Mr. Cecil Rowntree, M.B., B.S., F.R.C.S., has been appointed surgeon to St. Barnabas Home, Lloyd Square.

Dr. Percy Jakins has been appointed honorary laryngologist and aural surgeon to the National Children's Home and Orphanage, Bonner Road, and Harpenden.

The following appointments have been made by the Seamen's Hospital Society at the *Dreadnought* Hospital, Greenwich: House physicians, J. H. Lloyd, M.B., B.S., D. M. Morison, M.B., Ch.B.; house surgeons, C. E. Richardson, M.R.C.S., L.R.C.P., J. Renfrew White, M.B., B.S. And at the Albert Dock Hospital: House surgeon, Eric Marshall, M.R.C.S., L.R.C.P., D.T.M. (Eng.); house surgeon to out-patients, H. J. McCaw, M.B., Ch.B. Bernard G. Klein, M.B., B.Ch., has been appointed assistant pathologist at the London School of Clinical Medicine.

Mr. B. F. Beatson has been appointed junior assistant medical officer at Hanwell Mental Hospital.

The Rev. John Huxley, to whom a special tribute was paid at the recent annual meeting, has been re-appointed chaplain to the Norfolk and Norwich Hospital.

### MASSEUSE SISTER FOR CARDIFF HOSPITAL.

At the monthly meeting of the board of management of King Edward VII.'s Hospital, Cardiff, Nurse Higgins, of the Bristol Royal Infirmary, was unanimously appointed the new masseuse sister. Nurse Higgins was fourth in her year in passing the examination of the Incorporated Society of Trained Masseuses.

### Personalia.

Dr. J. H. Hertz, the Chief Rabbi, has been unanimously recommended by the House Committee to the next Court of Governors for election as a vice-president of the London Hospital.

Mr. A. L. Leon has been appointed to represent the London County Council at the Conference on Infant Mortality which is to be held in London in August next.

Mr. Cuthbert Wilkinson has been elected Chairman of the City of London Board of Guardians. Mr. Walter Bates has been elected Vice-Chairman.

Mr. A. Smith has resigned his post as dispenser at the Coventry and Warwickshire Hospital. The position of radiographer is now suggested as a separate post, and a fully qualified lady dispenser, to take the rank of sister and to help in the clerical work of the out-patient department, and a lady radiographer, who is also a properly trained masseuse and would undertake any electrical treatment, are likely to be appointed.

The Royal Army Medical Corps (T.F.) in Aberdeen is making great progress in hospital work and the nursing of the wounded. The second half of last winter's session was devoted to a course of training in this work, and the skill of the instructors as well as the enthusiasm of the students is indicated by the following awards made as a result of the examination held at the conclusion of the session: First Class Certificates of Honour and Prizes.—Staff Sergeant Stuart, Sergeant Gaskin, Sergeant-Major Walker, Sergeant Ross, Staff Sergeant Leslie, Private Low, Private Tastard, Sergeant Stephen, Private Ross, and Staff Sergeant Thorn. First Class Certificates of Honour.—Sergeant Cowie, Corporal Charles, Lance-Sergeant Russell. Second Class Certificates of Merit.—Sergeant Paterson, Private Black, Private Leith, Private Gillanders, Private Marr, Private Neave, Sergeant Brown, Sergeant-Piper Gilbert, Private Mutch, Lance-Sergeant Williamson, Privates Stephen, Aberdien, Bruce, Bain, Crighton, Wilson, Geddes, and Corporal Masson. At the conclusion of the ceremony Lieut.-Col. Kelly expressed his entire satisfaction with the standard of medical knowledge exhibited by members of the corps.

The Duke of Connaught, as Grand Prior of the Order of the Hospital of St. John of Jerusalem, at a special meeting of the Chapter-General, presented the service medal of the St. John Ambulance Brigade to the thirty-nine recipients, including District Surgeon E. B. Pooley, M.R.C.S., Corps Surgeon and Corps Superintendent J. Broomhead, M.B., Corps Surgeon R. Clegg, M.R.C.S., Corps Superintendent H. Bannister, and Corps Treasurer E. F. Millington.

### Gifts and Bequests.

The Royal Portsmouth Hospital is to receive a bequest of £1,000, less legacy duty, under the will of the late Mr. William Reed, of Southsea.

The President of the Royal Society has received letters from the Academy of Sciences of the Institute of France and the Faculty of Medicine of the University of Montpellier intimating donations of 500f. and 250f. respectively to the Lister Memorial Fund.

The Merchant Taylors' Company have voted a further grant of £1,000 to the funds of St. Bartholomew's Hospital.

In support of the presidency of Prince Arthur of Connaught at the centenary celebration in aid of the funds of the London Orphan Asylum, Watford, £210 has been given by the Merchant Taylors' Company.

Mr. William Johnston, of Salisbury, racehorse trainer and breeder, has left £100 each to the Salisbury Infirmary and the Fordingbridge Cottage Hospital.

The Highlands and Lowlands Para Rubber Company, Ltd., have sent to Mr. Austen Chamberlain a further contribution of £100 for the extension of the London School of Tropical Medicine.

### DOCTORS' WILLS.

The late Sir Thomas Frederick Chavasse, M.D., F.R.C.S., the eminent surgeon, of Birmingham, younger brother of the Bishop of Liverpool, left estate of the gross value of £17,407, of which £16,010 is net personalty.

Surgeon-General Sir Colvin Colvin-Smith, M.D., Hon. Surgeon to Queen Victoria, King Edward, and King George, an Indian Mutiny veteran, has left net personalty £10,072. The testator left his family portraits and the insignia of K.C.B. to his sons and their heirs in tail.

### A HOSPITAL FOUNDER'S DEATH.

There has passed away at the ripe age of ninety years, in the person of Mother Stanislaus Jones, the last of those Sisters of Mercy who, together with Miss Florence Nightingale, went as nurses to the Crimea in 1854. The deceased lady received in 1887 the Order of the Royal Red Cross from Queen Victoria, and died at the Hospital of St. John and Elizabeth, St. John's Wood, which she, with others, had been instrumental in founding.

